# Two New Species and a New Combination of the Subfamily Erythraeinae Based on Larval Stage (Acari: Trombidiformes: Erythraeidae) from China

**DOI:** 10.3390/insects13080706

**Published:** 2022-08-05

**Authors:** Si-Yuan Xu, Tian-Ci Yi, Jian-Jun Guo, Dao-Chao Jin

**Affiliations:** 1 Institute of Entomology, South Campus, Guizhou University, Guiyang 550025, China; 2 The Guizhou Provincial Key Laboratory for Plant Pest Management of Mountainous Region, Guiyang 550025, China; 3 The Scientific Observing and Experimental Station of Crop Pest in Guiyang, Ministry of Agriculture P. R. China, Guiyang 550025, China

**Keywords:** *Eatoniana*, *Erythraeus*, Oriental region, Palaearctic region, species key

## Abstract

**Simple Summary:**

Erythraeid mite members are large, larvae usually parasitize other arthropods, whereas the nymphs and adults are free-living predators of a small insect. Free-living adults/nymphs and parasitic larvae vary greatly in morphology, and classification is usually treated independently. To date, more than 850 species of erythraeid mites have been recorded. Among them, more than 580 species were described only as larvae. *Eatoniana* Cambridge, 1898 is a small genus within Erythraeidae comprising, 10 valid species worldwide, three species of which were only reported based on larvae. *Erythraeus* Latreille, 1806 includes 123 species distributed worldwide, with 69 species reported based on larvae alone. Here, two new species, *Eatoniana nanlingensis* Xu and Jin **sp. nov.** from Guangdong Province and *Erythraeus* (*Erythraeus*) *kunyuensis* Xu and Jin **sp. nov.** from Shandong Province, are described based on larval stage. We believe that the study will contribute to further studies on the taxonomy and phylogeny of Erythraeidae.

**Abstract:**

The species *Eatoniana yangshuonicus* (Haitlinger) **comb. nov.** is transferred from the genus *Erythraeus* to *Eatoniana* based on the basifemoral setal formula 2-2-1. Two new species, *Eatoniana nanlingensis* Xu and Jin **sp. nov.** and *Erythraeus* (*Erythraeus*) *kunyuensis* Xu and Jin **sp. nov.** are described and illustrated based on larvae. *Eatoniana* *nanlingensis* **sp. nov.** from the Oriental region (Guangdong Province), *Er*. (*Er*.) *kunyuensis* **sp. nov.** from the Palaearctic region (Shandong Province). An updated key to larval species of the genus *Eatoniana* of the world is presented.

## 1. Introduction

The subfamily Erythraeinae Robineau-Desvoidy contains 26 genera [1], 5 of which have been reported in China as follows: *Claverythraeus* Trägårdh, 1937 (Monotypic genus) based on active postlarval forms; *Eatoniana* Cambridge, 1898 based on larva; *Erythraeus* Latreille, 1806 (with two subgenera) based on larva or active postlarval forms; *Neophanolophus* Shiba, 1976 based on larva; and *Podosmaridia* Trägårdh, 1937 (Monotypic genus) based on active postlarval forms [2,3]. 

Mąkol and Sevsay [4] proposed that the genus *Abalakeus* Southcott, 1994 is a synonym of the genus *Eatoniana* Cambridge, 1898, and evidence was obtained through the reared individuals. The genus *Eatoniana* includes 10 species, one of which is a fossil species, *E*. *crinita* Sidorchuk, Konikiewicz, Welbourn and Mąkol, 2019 based on active postlarval instars [5], of the remaining 9 species, 3 are reported based on larvae only, 4 on active postlarval instars only and 2 on both larvae and active postlarval instars, respectively [2,4,5]. To date, only one species (not including the new combination proposed here, see below), *E*. *bambusae* (Zhang, 2000), has been reported from Fujian Province belong to the Oriental region in China, the Oriental region distribution of *Eatoniana* larvae species was missed by Mąkol and Sevsay [4].

The genus *Erythraeus* Latreille, 1806 comprises three subgenera, *Erythraeus* Latreille, 1806, *Parerythraeus* Southcott, 1946 and *Zaracarus* Southcott, 1995. The subgenus *Erythraeus* s. str. and the subgenus *Zaracarus* were described from adults or/and juveniles, respectively, the subgenus *Parerythraeus* was described from the active postlarval instars only [2,6,7,8]. Hitherto, seven species of the genus *Erythraeus* were described from China, one of them was based on active postlarval instars—*Erythraeus* (*Erythraeus*) *jacoti* Goosmann, 1925—while the other six species were based on their larval stage, four of which belong to subgenus *Erythraeus*—*Er*. (*Er*.) *chinensis* (Zheng, 2002); *Er.* (*Er.*) Xu, Yi, Guo and Jin, 2019; *Er*. (*Er*.) *yangshuonicus* Haitlinger, 2006 (being *Eatoniana yangshuonicus* **comb. nov**, see below); and *Er*. (*Er*.) *zhangi* Haitlinger, 2006, and two belong to subgenus *Zaracarus*—*Er*. (*Zaracarus*) *hainanensis* Xu, Yi, Guo and Jin, 2019 and *Er*. (*Z*.) *plumatus* Beron, 2008 [7].

In this study, *Eatoniana nanlingensis* **sp. nov.** and *Erythraeus* (*Erythraeus*) *kunyuensis* **sp. nov.** are described and illustrated based on larvae, collected from insects (Psocoptera) in Guangdong Province and herbaceous plants in Shandong Province, respectively. An updated key to the larvae of the *Eatoniana* species, known over the world, is presented.

## 2. Materials and Methods

Psocopteran insects and the mite larvae off the herbaceous plants were collected by an insect net in the field and subsequently preserved in small specimen vials containing 75% ethanol. Erythraeid larval specimens on psocopteran insects were detached by a fine brush under a stereomicroscope. Then, the larval specimens were prepared with Oudemans’ fluid and mounted in Hoyer’s medium. Figures were drawn with the aid of a drawing tube attached to a Nikon Eclipse Ni-E microscope. The terminology and abbreviations are adapted from Haitlinger and Saboori [9], Zhang and Goldarazena [10] and Xu et al. [7]. Measurements are expressed in micrometers (μm). The SD, standard deviation, keeps two decimal fractions.

## 3. Results

### 3.1. New Combination

urn:lsid:zoobank.org:pub:31DF2AC1-7FE8-4E09-99BA-9B57D70B4700

*Eatoniana* Cambridge, 1898

*Eatoniana yangshuonicus* (Haitlinger, 2006) **comb. nov.**

*Erythraeus* (*E*.) *yangshuonicus* Haitlinger, 2006: 86.

**Distribution.** Oriental region (Yangshuo County, Guilin City, Guangxi Province, China).

**Remark** **1.** Palpfemur and palpgenu, each with one seta; fn BFe = 2-2-1; fn TFe = 5-5-5; fnTr = 1-1-1; fnCx = 1-1-1 in *Eatoninan yangshounicus* (Haitlinger, 2006), according to the larval diagnosis of *Eatoniana* by Mąkol and Sevsay [4] and key to world genera of larval Erythraeinae by Noei et al. [1,11], this species is transferred from *Erythraeus* (*Erythraeus*) to *Eatoniana*.

From the identification keys to *Eatoniana* species by Mąkol and Sevsay [4] and Noei and Rabieh [12], *E*. *yangshuonicus* **comb. nov.** is relative to *E*. *plumipes* (L. Koch, 1856). *Eatoniana yangshuonicus* **comb. nov.** differs from *E*. *plumipes* by scutum shape (trapezoidal vs. oval or pentagonal), the longer Ti I (346 vs. 169–235), Ti II (332 vs. 158–203) and Ti III (496 vs. 220–302).

### 3.2. New Species

**Figure 1:**
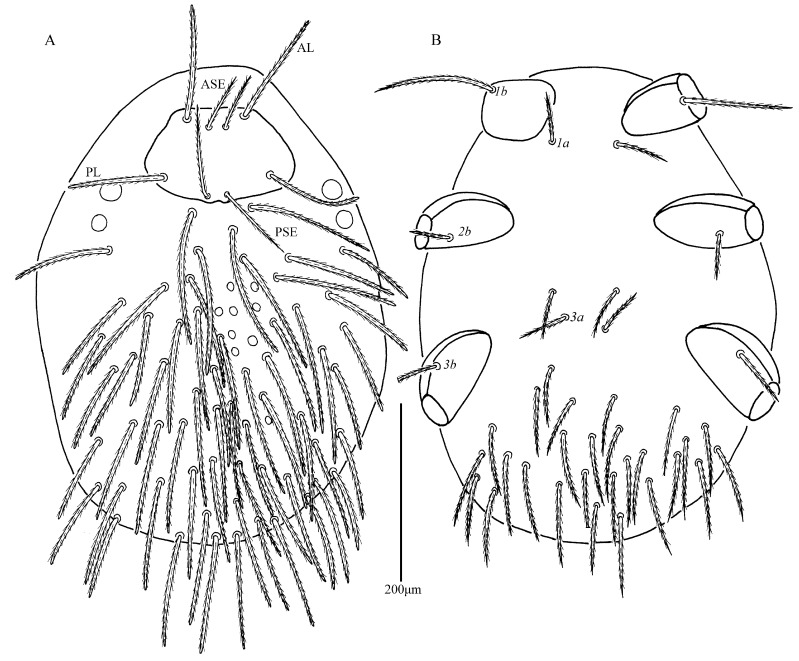
*Eatoniana nanlingensis* **sp. nov.**, larva. (**A**). Dorsal view of idiosoma. (**B**). Ventral view of idiosoma.

**Figure 2:**
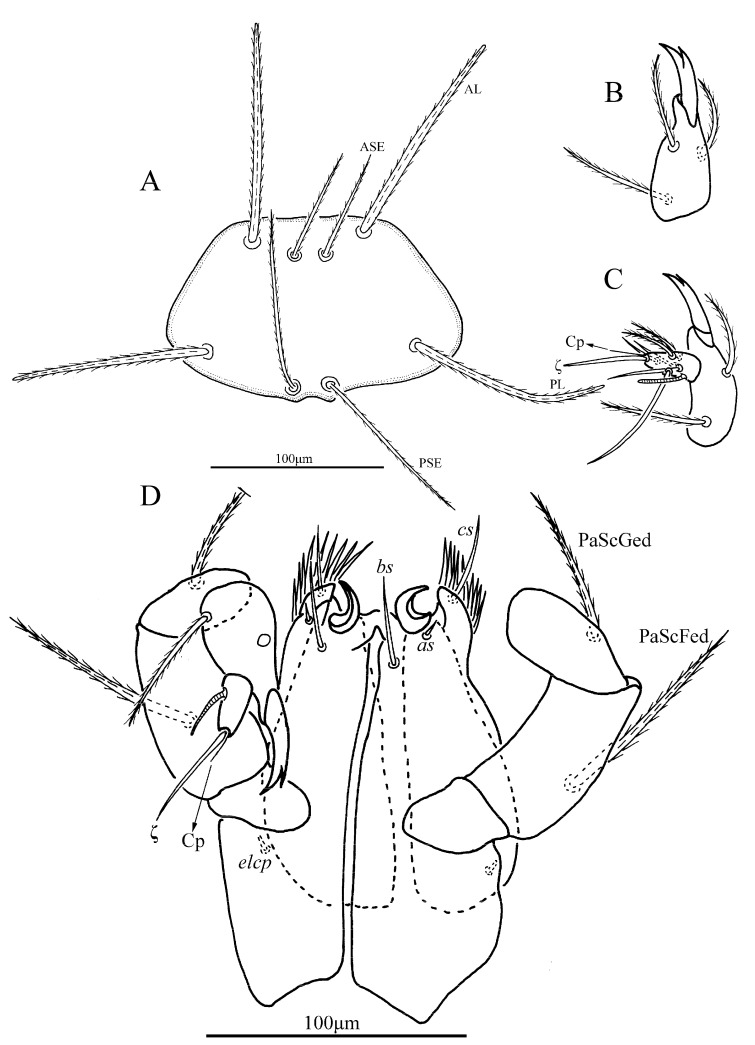
*Eatoniana nanlingensis***sp. nov.**, larva. (**A**). Scutum. (**B**). Dorsal view of palp tibia. (**C**). Ventral view of palp tarsus. (**D**). Ventral view of gnathosoma.

**Figure 3:**
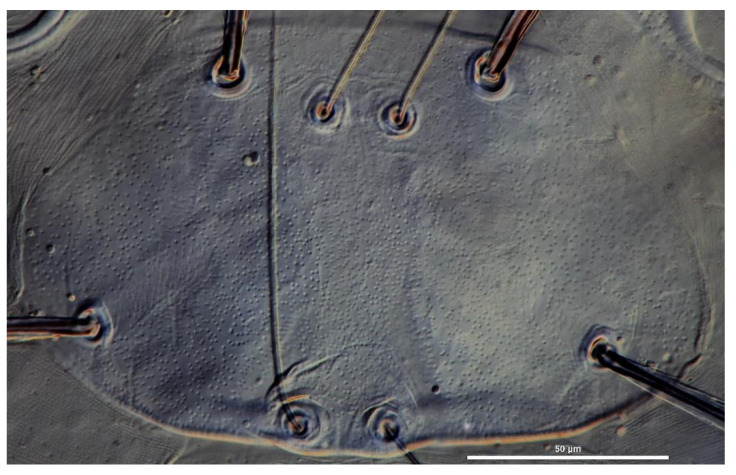
*Eatoniana nanlingensis* **sp. nov.**, larva. Photograph. Showing outline of scutum.

**Figure 4:**
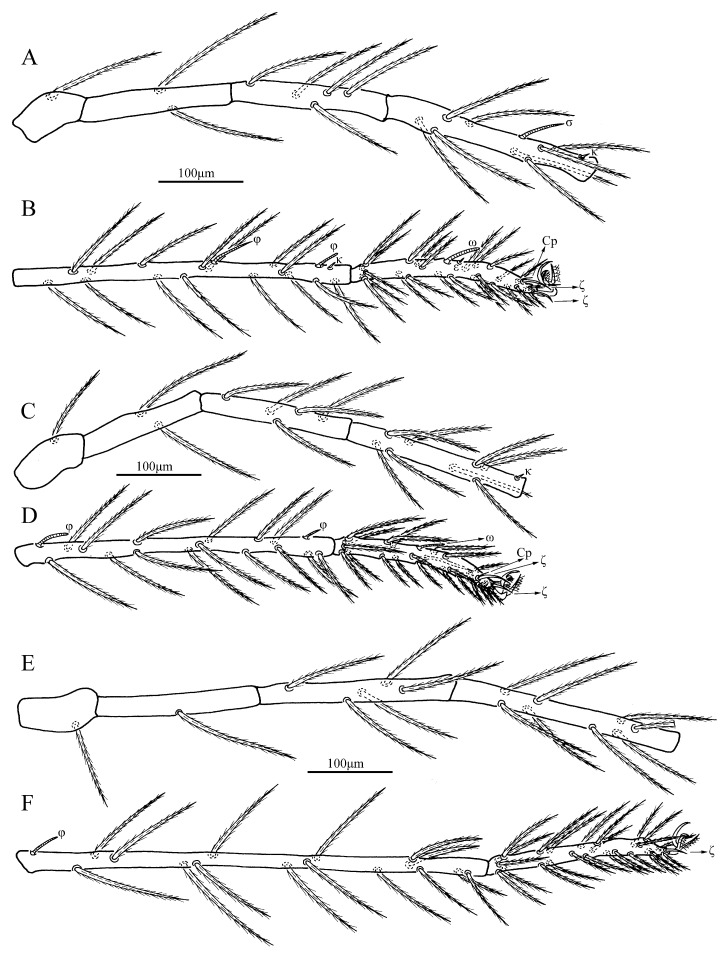
*Eatoniana nanlingensis***sp. nov.**, larva. (**A**). Leg I, trochanter—genu. (**B**). Leg I, tibia—tarsus. (**C**). Leg II, trochanter—genu. (**D**). Leg II, tibia—tarsus. (**E**). Leg III, trochanter—genu. (**F**). Leg III, tibia—tarsus.

*Eatoniana nanlingensis* Xu and Jin **sp. nov.** (Figure 1, Figure 2, Figure 3 and Figure 4)

urn:lsid:zoobank.org:act:9199505D-4BFF-4BB2-A682-11ED5347CEF1

**Diagnosis (larva).** Sensillary setae (ASE and PSE) both fully barbed; fD 68; Ti I 392–393; Ti III 547–549; fn Ti = 15-15-15. 

**Description.** Dorsum. Idiosoma almost oval, with 68 barbed setae (fD = 68 in paratype) (Figure 1A). Two pairs of eyes without platelets, posterolateral to scutum. Scutum about trapezoidal with rounded angles, anterior margin and lateral margin almost straight, posterior margin slightly cambered with small concave portion between bases of PSE (Figure 2A and Figure 3). Scutum with two pairs of entirely barbed sensilla (ASE and PSE) and scutalae (AL and PL). PSE much longer than ASE, AL slightly longer than PL (Table 1). 

Venter. All ventral setae, including coxalae, setiform, barbed and with pointed tips (Figure 1B). Two pairs of intercoxal setae (*1a* and *3a*), *1a* slightly longer than *3a* (Table 1); *1a* located between and slightly posterior to coxae I, *3a* almost in a line with anterior edges of coxae III; behind coxae III with 27 setae (28 in paratype), two setae (55–56) between coxae II and III. Three pairs of coxalae (*1b*, *2b* and *3b*), *1b* much longer than *3b*, *3b* longer than *2b* (Table 1). Length of setae increasing from 67 posterior to *3a* to 91 near posterior margin.

Gnathosoma. With one pair of nude galealae (*cs*), two pairs of nude hypostomalae (*as* and *bs*), hypostomal lip fimbriate (Figure 2D); *bs* much longer than *as* (Table 1). Dorsum of palpfemur and palpgenu each with one barbed and pointed seta (PaScFed and PaScGed), PaScFed longer than PaScGed; palptibia with three barbed setae, two on the venter, odontus bifid (Figure 2B–D). Palptarsus with eight setae including two barbed setae, four nude setae, one solenidion (ω), one eupathidium (ζ) and one Cp (Figure 2C), fPp = 0-B-B-3B_2_-2B4NωζCp. Palpal supracoxal seta (elcp) peg-like (Figure 2D).

Legs (Figure 4). With seven segments (femora divided). IP = 4283–4325 (Holotype and paratype). Dorsum of coxa I with a supracoxal seta (*eI*) which is peg-like with a rounded tip. Anterior claw feather-like with distal hook, posterior claw feather-like without distal hook and claw-like empodium falciform. Normal setae on legs barbed and pointed. Leg setal formula: leg I: Cx—1n; Tr—1n; Bfe—2n; Tfe—5n; Ge—1σ, 1κ, 8n; Ti—2φ, 1κ, 15n; Ta—1ω, 1ε, 2ζ, 1Cp, 24n. leg II: Cx—1n; Tr—1n; Bfe—2n; Tfe—5n; Ge—1κ, 8n; Ti—2φ, 15n; Ta—1ω, 2ζ, 1Cp, 23n. leg III: Cx—1n; Tr—1n; Bfe—1n; Tfe—5n; Ge—8n; Ti—1φ, 15n; Ta—1ζ, 24n. Morphometric data of legs are listed in Table 1.

**Etymology.** The name of the new species is derived from the Nanling National Natural Reserve where it was collected.

**Types.** Holotype, larva, unidentified Psocoptera (Insect), collected by Si-Yuan Xu on 29 April 2019, from Nanling National Natural Reserve (Altitude: 1013 m), Guangdong Province, China. Paratype, one larva, unidentified Psocoptera (Insect), collected by Si-Yuan Xu on 29 April 2019, from Nanling National Natural Reserve (Altitude: 971 m), Guangdong Province, China.

The holotype and paratype are deposited in the Institute of Entomology, Guizhou University, Guiyang, China (GUGC). 

**Distribution.** China: Guangdong Province.

**Remark** **2.** Hitherto, a total of six species in the genus *Eatoniana* were described based on larvae [2,4,12,13,14,15,16]: *E*. *bambusae* (Zhang, 2000) from China; *E*. *chekei* (Southcott, 1994) from Niger; *E*. *gonabadensis* (Ahmadi, Hajiqanbar and Saboori, 2012) from Iran; *E*. *lorestanicus* (Saboori and Lanhinani, 2003) from Iran and Spain; *E*. *plumipes* (L. Koch, 1856) from Algeria, Egypt, France, Great Britain, Greece, Iran, Mauritania, Spain, Tunisia and Turkey. The Iranian records were larvae, the Turkish records were larvae and active postlarval instars and the Chinese reports including *E*. *yangshuonicus* (Haitlinger, 2006) **comb. nov.** were larvae.

*Eatoniana nanlingensis* **sp. nov.** can be easily separated from *E*. *chekei*, *E*. *gonabadensis* and *E*. *lorestanicus* based on TFe I without a solenidion (vs. present); the new species can be easily separated from *E*. *plumipes* and *E*. *yangshuonicus* based on eyes without platelets (vs. present) and two setae present between coxae II and III (vs. absence).

*Eatoniana nanlingensis* **sp. nov.** differs from *E*. *bambusae* by scutum shape (trapezoidal vs. pentagonal), the shape of ASE and PSE (having fully fine barbs vs. having fine barbs on distal halves), the presence of eupathidium on Ta III (vs. absence), fD (68 vs. 50), fn Ti (15-15-15 vs. 15-15-14), fn Ta (24-23-24 vs. 27-23-25) and the number and shape of palptarsus normal setae (six setae (2B4N) vs. five setae (5N)).

Key to *Eatoniana* larvae of the world (Updated from Noei and Rabieh (2019))

1. Solenidion on TFe I absent; anterior claw on Ta I–III feather-like and with or without distal hook; posterior claw on Ta I–III feather-like.......................2

–. Solenidion on TFe I present; anterior claw on Ta I–III simple, falciform; posterior claw on Ta I–III with spoon-like rod and covered with numerous onychotrichs....................5

2. Between coxae II and III without seta; eyes with platelets....................3

–. Between coxae II and III with two setae; eyes without platelets......................4

3. Scutum shape oval or pentagonal; Ti I = 169–235; Ti II = 158–203; Ti III = 220–302....................*E. plumipes* (L. Koch, 1856)

–. Scutum shape trapezoidal; Ti I = 346; Ti II = 332; Ti III = 496.......................*E*. *yangshuonicus* **comb. nov.** (Haitlinger, 2006)

4. Scutum shape pentagonal; fD = 50; eupathidium absent on Ta III............................*E*. *bambusae* (Zhang, 2000)

–. Scutum shape trapezoidal; fD = 68; eupathidium present on Ta III...........................*E*. *nanlingensis* **sp. nov.**

5. Solenidion on Ge III absent........................*E*. *chekei* (Southcott, 1994)

–. Solenidion on Ge III present.......................6

6. Two pairs of setae between coxae III; Ti III = 262–301, IP = 2708–2871....................... * E*. *lorestanicus* (Saboori & Lachinani, 2003)

–. One pair of setae between coxae III; Ti III = 118–179, IP = 1893–2015........................ * E*. *gonabadensis* (Ahmadi, Hajiqanbar and Saboori, 2012)

*Erythraeus* Latreille, 1806

**Figure 5:**
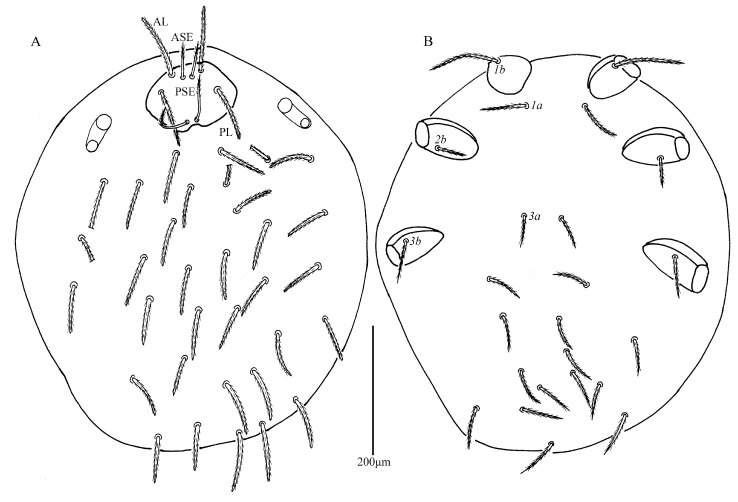
*Erythraeus* (*Erythraeus*) *kunyuensis* **sp. nov.**, larva. (**A**). Dorsal view of idiosoma. (**B**). Ventral view of idiosoma.

**Figure 6:**
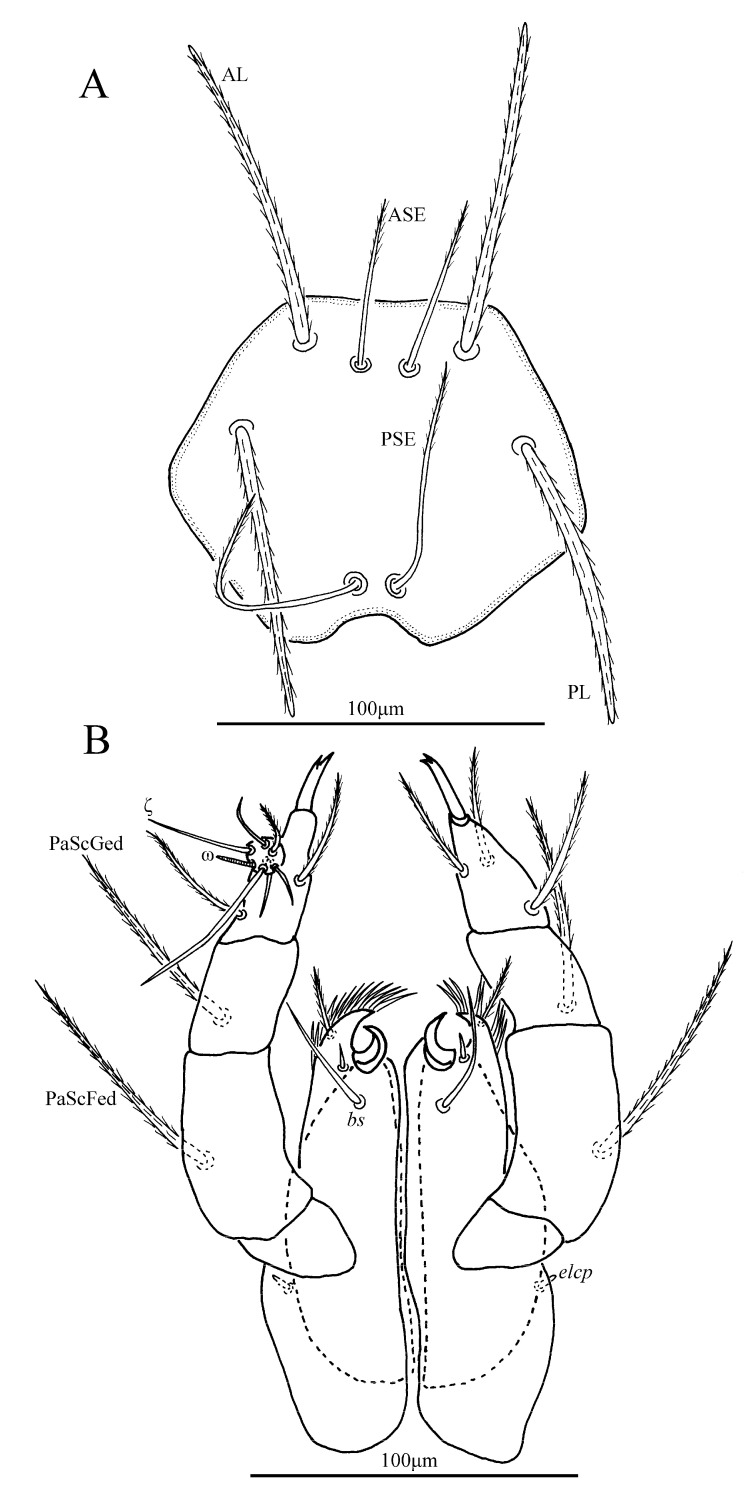
*Erythraeus* (*Erythraeus*) *kunyuensis* **sp. nov.**, larva. (**A**). Scutum. (**B**). Ventral view of gnathosoma.

**Figure 7:**
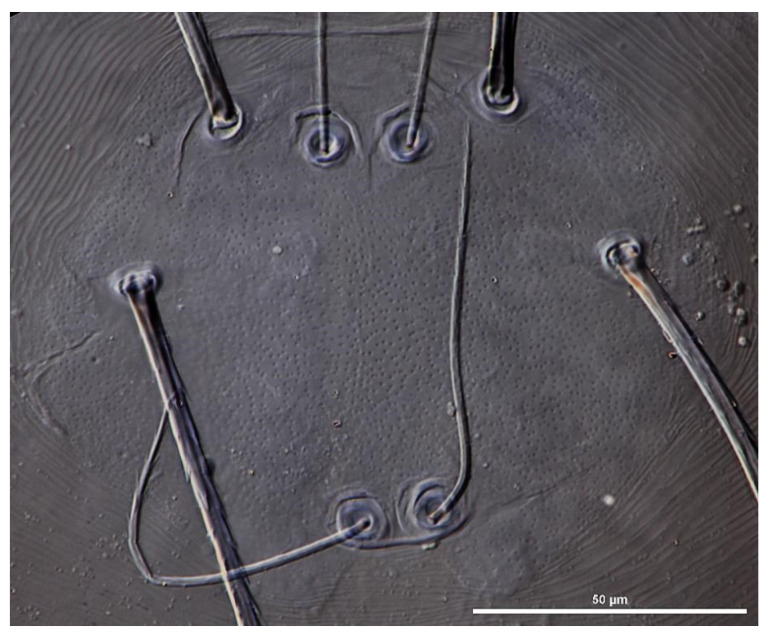
*Erythraeus* (*Erythraeus*) *kunyuensis* **sp. nov.**, larva. Photograph. Showing outline of scutum.

**Figure 8:**
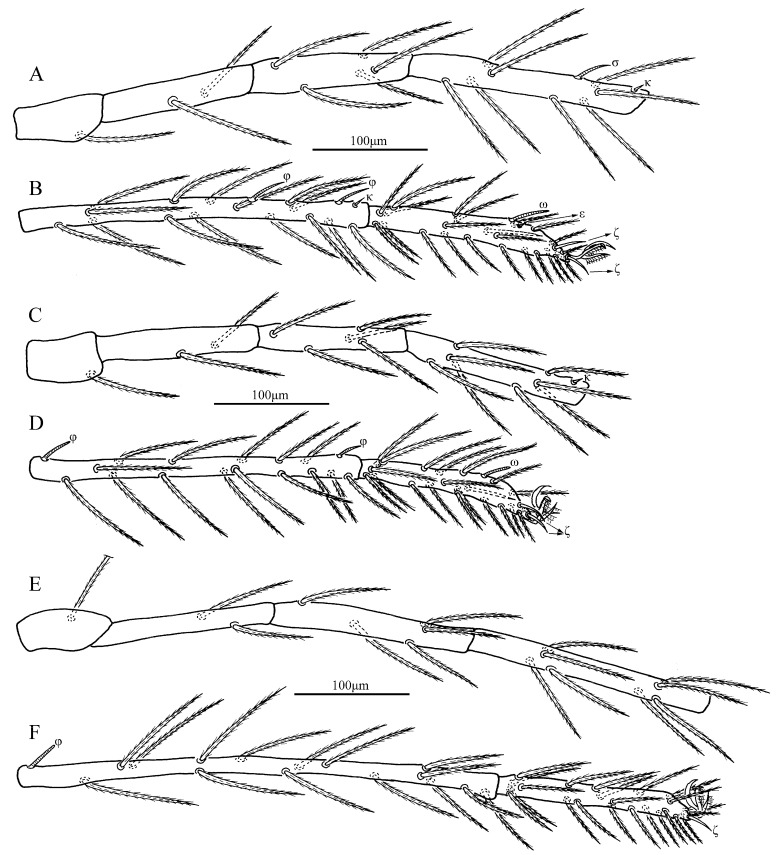
*Erythraeus* (*Erythraeus*) *kunyuensis* **sp. nov.**, larva. (**A**). Leg I, trochanter—genu. (**B**). Leg I, tibia—tarsus. (**C**). Leg II, trochanter—genu. (**D**). Leg II, tibia—tarsus. (**E**). Leg III, trochanter—genu. (**F**). Leg III, tibia—tarsus.

*Erythraeus* (*Erythraeus*) *kunyuensis* Xu and Jin **sp. nov.** (Figure 5, Figure 6, Figure 7 and Figure 8)

urn:lsid:zoobank.org:act:B9C1AA9D-830F-411D-9D22-32652D26C3E9

**Diagnosis (larva).** Eyes with platelets; Sensillary setae (ASE and PSE) with barbs on distal halves; fD 32–33; Ti I 298–310; Ti III 409–449; fn BFe = 2-2-2. 

**Description.** Dorsum. Idiosoma almost oval or sub-circular, with 33 barbed setae (fD = 32–33 in paratypes) (Figure 5A). Two pairs of eyes present on platelets. Scutum is pentagonal, anterior margin and anterolateral margin almost straight and posterolateral margin is almost straight with visible concavity between the base of PSE (Figure 6A and Figure 7). Scutum with two pairs of sensilla (ASE and PSE) and scutalae (AL and PL). ASE and PSE with fine barbs on distal half, PSE much longer than ASE. AL longer than PL, both entirely barbed (Table 2).

*Venter*. All ventral setae, including coxalae, setiform, barbed and with pointed tips (Figure 5B). Two pairs of intercoxal setae (*1a* and *3a*) present, *2a* absent, *1a* longer than *3a*; Three pairs of coxalae (*1b*, *2b* and *3b*), *1b* much longer than *3b* and *2b*, *3b* longer than *2b* (Table 2). Intercoxal setae (*1a* and *3a*) located between coxae I and coxae III, respectively, *1a* located between and posterior to coxae I, *3a* almost in a line with anterior edges of coxae III; behind coxae III with 14 setae (14 in paratypes). 

*Gnathosoma*. With one pair of barbed galealae (*cs*), two pairs of nude hypostomalae (*as* and *bs*), hypostomal lip fimbriate (Figure 6B); *bs* much longer than *as* (Table 2). Dorsum of palpfemur and palpgenu each with one barbed and pointed seta (PaScFed and PaScGed), PaScFed slightly longer than PaScGed; palptibia with two barbed setae on the venter, one barbed seta on dorsal, odontus bifid (Figure 6B). Palptarsus with seven setae including one barbed seta, four nude setae, one solenidion (ω), one eupathidium (ζ), fPp = 0-B-B-3B_2_-B4Nωζ. Palpal supracoxal seta (*elcp*) peg-like (Figure 6B).

*Legs* (Figure 8). With seven segments (femora divided). IP = 3367–3505 (Holotype and three paratypes). Dorsum of coxa I with a supracoxal seta (*eI*) which is peg-like and rounded at the tip. Normal setae on legs are barbed and pointed. Leg setal formula: Leg I: Cx—1n; Tr—1n; Bfe—2n; Tfe—5n; Ge—1σ, 1κ, 8n; Ti—2φ, 1κ, 15n; Ta—1ω, 1ε, 2ζ, 24n. Leg II: Cx—1n; Tr—1n; Bfe—2n; Tfe—5n; Ge—1κ, 8n; Ti—2φ, 15n; Ta—1ω, 2ζ, 21n. Leg III: Cx—1n; Tr—1n; Bfe—2n; Tfe—5n; Ge—8n; Ti—1φ, 15n; Ta—1ζ, 23n. Morphometric data of legs are listed in Table 2.

**Etymology.** The name of the new species is derived from the National Natural Reserve where it was collected.

**Types.** Holotype, larva, unidentified herbaceous plants, collected by Si-Yuan Xu on 21 July 2018, from Kunyushan National Natural Reserve (Altitude: 135 m), Shandong Province, China. Paratypes, three larvae, the same data as the holotype.

The holotype and three paratypes are deposited in the Institute of Entomology, Guizhou University, Guiyang, China (GUGC). 

**Distribution.** China: Shandong Province.

**Remark** **3.** *Erythraeus* (*Erythraeus*) *kunyuensis* **sp. nov.** belongs to the species group with fn BFe 2-2-2 (2-2-1). This group includes 10 species [7,17,18,19,20,21,22,23,24]: *Er*. (*Er*.) *aphidivorous* Šundić, Haitlinger, Michaud and Colares, 2015; *Er.* (*Er*.) *chinensis* (Zheng, 2002); *Er*. (*Er*.) *etnaensis* Haitlinger, 2011; *Er*. (*Er*.) *hubeiensis* Xu, Yi, Guo and Jin, 2019; *Er*. (*Er*.) *kacperi* Haitlinger, 2004 (fn BFe 2-2-1); *Er*. (*Er*.) *phalangoides* (De Geer, 1778); *Er*. (*Er*.) *picaforticus* Haitlinger, 2002; *Er*. (*Er*.) *serbicus* Šundić, Haitlinger and Hakimitabar, 2015; *Er*. (*Er*.) *tinnae* Haitlinger, 1997; *Er*. (*Er*.) *walii* Kamran, Afzal, Raza, Bashir and Ahmad, 2011.

In the original description, Haitlinger [25] mentioned *Er*. (*Er.*) *kacperi* with only one seta in BFe III, but figured it with two setae (Figure 8). Moreover, Haitlinger [25] figured *Er*. (*Er.*) *kacperi* with only one pair of eyes (Figure 1), not mentioned in the text, while all other known *Erythraeus* members have two pairs of eyes. Therefore, it seems that the taxonomic status of *Er*. (*Er.*) *kacperi* needs to be further studied.

*Erythraeus* (*Erythraeus*) *kunyuensis* **sp. nov.** differs from *Er*. (*Er*.) *aphidivorous* due to the shape of *2b* and *3a* (pointed tips vs. bifid at the end), longer *1b* (111–120 vs. 92–101), *3a* (53–61 vs. 35–38), Ti I (298–310 vs. 278), Ti II (282–304 vs. 266–269), shorter L (104–107 vs. 117–125) and W (129–137 vs. 157–163).

*Er*. (*Er*.) *kunyuensis***sp. nov.** differs from *Er.* (*Er*.) *chinensis* due to the shape of the scutum (pentagonal vs. oval), the shape of ASE and PSE (with barbs on distal halves vs. nude), number of hypostomalae (two pairs vs. one pair), Ti I without companion seta (vs. present), apices of *2b* and *3b* pointed (vs. blunt), smaller number of setae in fD (32–33 vs. 40), longer *1b* (111–120 vs. 91), *2b* (40–47 vs. 27) and *3b* (63–66 vs. 38).

*Er*. (*Er*.) *kunyuensis***sp. nov.** differs from *Er.* (*Er*.) *etnaensis* in having Ge I and Ge II with microsetae (vs. absent), smaller number of setae in fD (32–33 vs. 64), shorter W (129–137 vs. 150), PW (83–92 vs. 110), AP (34–39 vs. 58), longer ISD (70–79 vs. 54), ASE (49–55 vs. 38), Ti I (298–310 vs. 262) and IP (3367–3505 vs. 3332).

*Er*. (*Er*.) *kunyuensis***sp. nov.** differs from *Er*. (*Er*.) *hubeiensis* due to the shape of the scutum (pentagonal vs. sub-rounded), Ti I without companion seta (vs. present), BFe I with two barbed setae (vs. one barbed seta and one nude seta), eyes are with platelets (vs. without platelets), longer *3b* (63–66 vs. 41–47), W (129–137 vs. 112–123), PaScFed (70–76 vs. 47–59) and GL (136–147 vs. 121–130).

*Er*. (*Er*.) *kunyuensis***sp. nov.** differs from *Er*. (*Er*.) *kacperi* by having Ge I with one solenidion (vs. absent), longer DS (61–91 vs. 38–54), *1b* (111–120 vs. 88), L (104–107 vs. 90), W (129–137 vs. 110), Ti I (298–310 vs. 184), Ti II (282–304 vs. 180) and Ti III (409–449 vs. 280).

*Er*. (*Er*.) *kunyuensis***sp. nov.** differs from *Er*. (*Er*.) *phalangoides* due to the shape of the scutum (pentagonal vs. oval), longer *1b* (111–120 vs. 80–100), *2b* (40–47 vs. 25–38), *3b* (63–66 vs. 28–40), ASE (49–55 vs. 14–32), PaScFed (70–76 vs. 40–50), Ti I (298–310 vs. 98–141), Ti II (282–304 vs. 97–131) and Ti III (409–449 vs. 145–200).

*Er*. (*Er*.) *kunyuensis***sp. nov.** differs from *Er*. (*Er*.) *picaforticus* due to the longer Ti I (298–310 vs. 214), Ti II (282–304 vs. 224), Ti III (409–449 vs. 362), shorter AW (48–54 vs. 84), PW (83–92 vs. 136), W (129–137 vs. 190) and fD (32–33 vs. 72).

*Er*. (*Er*.) *kunyuensis***sp. nov.** differs from *Er*. (*Er*.) *serbicus* as Ta II has no famulus (vs. with famulus), the shape of the scutum (pentagonal vs. oval), smaller number of setae in fD (32–33 vs. 70–71), longer Ti I (298–310 vs. 175–190), Ti II (282–304 vs. 180–192) and Ti III (409–449 vs. 261–274).

*Er*. (*Er*.) *kunyuensis***sp. nov.** differs from *Er*. (*Er*.) *tinnae* due to the shape of *1a* (barbed vs. nude), fD (32–33 vs. 47), longer ASE (49–55 vs. 36), shorter L (104–107 vs. 132), W (129–137 vs. 194), AW (48–54 vs. 80), PW (83–92 vs. 144) and PSE (70–75 vs. 92).

*Er*. (*Er*.) *kunyuensis***sp. nov.** differs from *Er*. (*Er*.) *walii* by solenidion on Ge II is absent (vs. present), longer DS (61–91 vs. 32–43), *1b* (111–120 vs. 71–76), L (104–107 vs. 77–83), ASE (49–55 vs. 26–28), PaScFed (70–76 vs. 50–51), Ti I (298–310 vs. 173–176), Ti II (282–304 vs. 158–163) and Ti III (409–449 vs. 248–251).

## 4. Discussion

The genus *Eatoniana* Cambridge, 1898 belongs to the subfamily Erythraeinae (Trombidiformes: Erythraeidae). Based on previously published data and the present study, there are 12 species of this genus [2,4,5], 3 of which are from the Oriental region in China [14,16]: *E*. *bambusae* (Zhang, 2000) from Fujian Province, *E*. *yangshuonicus* (Haitlinger, 2006) **comb. nov.** from Guangxi Province and *E*. *nanlingensis* **sp. nov.** from Guangdong Province. Among the remaining nine species, five are from the Palaearctic region (*Earoniana halleri* (Banks, 1900) from Switzerland, *E*. *plumifer* (Birula, 1893) from Armenia and Turkmenistan, *E*. *jahromiensis* (Sedghi, Saboori and Hakimitabar, 2010) from Iran, *E*. *lorestanicus* (Saboori and Lachinani, 2003) from Iran and Spain, *E*. *crinita* Sidorchuk, Konikiewicz, Welbourn & Mąkol, 2019 from Russia (Kaliningrad region) and Eocene Baltic amber), two are from the Afrotropical region (*E*. *namaquensis* (Lawrence, 1937) from South Africa, *E*. *chekei* (Southcott, 1994) from Niger), one is from the Neotropical region (*E*. *claviger* (Berlese, 1916) from Argentina) and one is recorded in both the Palaearctic and Afrotropical regions (*E*. *plumipes* (L. Koch, 1856) in Algeria, Egypt, France, Great Britain, Greece, Iran, Spain, Tunisia, Turkey and Mauritania) [2,4,5]. Zoogeographically, the known species of the genus *Eatoniana* are mainly reported from the Palaearctic region. China straddles two zoogeographic regions, Palaearctic and Oriental, but all three known species of this genus are from the Oriental region. Therefore, the species richness of this genus in the Palaearctic region of China still needs to be investigated.

The genus *Erythraeus* Latreille, 1806 (Erythraeidae: Erythraeinae) has been reported on all continents except Antarctica [7], this genus includes 123 species distributed worldwide, with 69 species reported based on larvae only [6,8]. In China, four species of this genus are from the Oriental region, *Erythraeus* (*Erythraeus*) *chinensis* (Zheng, 2002) from Hunan Province, *Er*. (*Er*.) *hubeiensis* Xu, Yi, Guo and Jin, 2019 from Hubei Province, *Er*. (*Zaracarus*) *plumatus* Beron, 2008 from Taiwan Province and *Er*. (*Z*.) *hainanensis* Xu, Yi, Guo and Jin, 2019 from Hainan Province, and three from the Palaearctic region, the collection site of *Er*. (*Er*.) *jacoti* Goosmann, 1925 is near Beijing municipality, *Er*. (*Er*.) *zhangi* Haitlinger, 2006 from Beijing municipality and *Er*. (*Er*.) *kunyuensis* **sp. nov.** from Shandong Province. Based on the data of previous literature and this study, only seven species of *Erythraeus* were reported in China [2,7], which is less than 6% of the known *Erythraeus* species in the world. It is undoubtedly necessary to continue the investigation and collection of the two and other genera of the family Erythraeidae to determine their diversity in China.

## Figures and Tables

**Table 1:** Measurements of *Eatoniana nanlingensis* **sp. nov.** (larva, a = paratype).

Character	Holotype	a	SD	Range	Character	Holotype	a	SD	Range
fD	68	68	0	68–68	Tr I	88	80	4	80–88
fV	27	28	0.5	27–28	Cx I	77	82	2.5	77–82
NDV	95	96	0.5	97–98	Ta II (H)	24	21	1.5	21–24
IL	457	537	40	457–537	Ta II (L)	194	200	3	194–200
IW	382	426	22	382–426	Ti II	361	376	7.5	361–376
DS	86–144	86–153	0.00–4.50	86–153	Ge II	182	196	7	182–196
PDS	94–136	89–132	2.00–2.50	89–136	TFe II	176	172	2	172–176
Oc1	20	22	1	20–22	BFe II	159	163	2	159–163
Oc2	19	18	0.5	18–19	Tr II	86	74	6	74–86
*1a*	60	62	1	60–62	Cx II	98	103	2.5	98–103
*3a*	58	56	1	56–58	Ta III (H)	18	20	1	18–20
*1b*	133	130	1.5	130–133	Ta III (L)	216	219	1.5	216–219
*2b*	50	49	0.5	49–50	Ti III	547	549	1	547–549
*3b*	70	66	2	66–70	Ge III	271	267	2	267–271
L	102	105	1.5	102–105	TFe III	224	231	3.5	224–231
W	165	173	4	165–173	BFe III	192	198	3	192–198
AW	63	67	2	63–67	Tr III	89	80	4.5	80–89
PW	116	126	5	116–126	Cx III	103	111	4	103–111
MA	25	26	0.5	25–26	Leg I	1385	1386	0.5	1385–1386
AA	18	19	0.5	18–19	Leg II	1256	1284	14	1256–1284
SB	21	23	1	21–23	Leg III	1642	1655	6.5	1642–1655
ISD	74	77	1.5	74–77	IP	4283	4325	21	4283–4325
AP	67	70	1.5	67–70	AL/PL	1.13	1.17	0.02	1.13–1.17
AL	126	127	0.5	126–127	AW/AP	0.94	0.96	0.01	0.94–0.96
PL	112	109	1.5	109–112	AW/ISD	0.85	0.87	0.01	0.85–0.87
ASE	63	60	1.5	60–63	AW/AL	0.5	0.53	0.01	0.50–0.53
PSE	100	104	2	100–104	L/W	0.62	0.61	0.01	0.61–0.62
*as*	7	8	0.5	7–8	L/ISD	1.38	1.36	0.01	1.36–1.38
*bs*	33	34	0.5	33–34	W/AW	2.62	2.58	0.02	2.58–2.62
*cs*	28	31	1.5	28–31	PW/AW	1.84	1.88	0.02	1.84–1.88
PaScFed	86	79	3.5	79–86	PW/L	1.14	1.2	0.03	1.14–1.20
PaScGed	61	57	2	57–61	ISD/AP	1.1	1.1	0	1.10–1.10
GL	168	164	2	164–168	Ti I/Ge I	1.55	1.57	0.01	1.55–1.57
Ta I (H)	23	20	1.5	20–23	Ti II/Ge II	1.98	1.92	0.03	1.92–1.92
Ta I (L)	213	220	3.5	213–220	Ti III/Ge III	2.02	2.06	0.02	2.02–2.06
Ti I	393	392	0.5	392–393	Ti I/AW	6.24	5.85	0.19	5.85–6.24
Ge I	253	249	2	249–253	Ti III/AW	8.68	8.19	0.24	8.19–8.68
TFe I	182	187	2.5	182–187	Ti III/Ti I	1.39	1.4	0	1.39–1.40
BFe I	179	176	1.5	176–179	Ti II/PW	3.11	2.98	0.06	2.98–3.11

**Table 2:** Measurements of *Erythraeus* (*Erythraeus*) *kunyuensis* **sp. nov.** (larvae, a–c = paratypes).

Character	Holotype	a	b	c	SD	Range	Character	Holotype	a	b	c	SD	Range
fD	33	33	32	33	0.43	32–33	Tr I	73	75	72	63	4.6	63–75
fV	14	14	14	14	0	14–14	Cx I	76	77	75	71	2.28	71–77
NDV	47	47	46	47	0.43	46–47	Ta II (H)	18	18	19	21	1.22	18–21
IL	635	383	658	389	130.52	383–658	Ta II(L)	151	157	156	155	2.28	151–157
IW	556	313	543	327	114.95	313–556	Ti II	282	298	289	304	8.41	282–304
DS	67–91	63–88	61–87	66–80	2.38–4.03	61–91	Ge II	156	158	163	165	3.64	156–165
PDS	72–91	69–88	73–87	72–80	1.50–4.03	69–91	TFe II	127	136	128	137	4.53	127–137
Oc1	21	20	23	21	1.09	20–23	BFe II	138	143	137	140	2.29	137–143
Oc2	16	17	17	15	0.83	15–17	Tr II	62	61	62	64	1.09	61–64
*1a*	72	65	71	63	3.83	63–72	Cx II	88	93	86	87	2.69	86–93
*3a*	56	61	53	57	2.86	53–61	Ta III (H)	18	19	17	18	0.71	17–19
*1b*	118	120	111	113	3.64	111–120	Ta III (L)	169	181	184	192	8.26	169–192
*2b*	47	40	43	43	2.49	40–47	Ti III	409	436	422	449	14.98	409–449
*3b*	64	66	/	63	1.25	63–66	Ge III	210	209	213	208	1.87	208–213
L	104	104	107	106	1.3	104–107	TFe III	168	172	166	173	2.86	166–173
W	129	137	133	136	3.11	129–137	BFe III	150	157	151	153	2.68	150–157
AW	49	48	54	50	2.28	48–54	Tr III	73	64	68	75	4.3	64–75
PW	87	83	92	91	3.56	83–92	Cx III	94	103	92	105	5.59	92–105
MA	19	21	19	19	0.87	19–21	Leg I	1090	1137	1093	1092	19.66	1090–1137
AA	15	18	16	14	1.48	14–18	Leg II	1004	1046	1021	1052	19.33	1004–1052
SB	13	16	15	13	1.3	13–16	Leg III	1273	1322	1296	1355	30.52	1273–1355
ISD	70	74	78	79	3.56	70–79	IP	3367	3505	3410	3499	58.79	3367–3505
AP	34	38	37	39	1.87	34–39	AL/PL	1.15	1.34	1.24	1.29	0.07	1.15–1.34
AL	102	111	105	108	3.35	102–111	AW/AP	1.44	1.26	1.46	1.28	0.09	1.26–1.46
PL	89	83	85	84	2.28	83–89	AW/ISD	0.7	0.65	0.69	0.63	0.03	0.63–0.70
ASE	53	55	49	53	2.18	49–55	AW/AL	0.48	0.43	0.51	0.46	0.03	0.43–0.51
PSE	72	74	70	75	1.92	70–75	L/W	0.81	0.76	0.8	0.78	0.02	0.76–0.81
*as*	9	11	9	8	1.09	8–11	L/ISD	1.49	1.41	1.37	1.34	0.05	1.34–1.49
*bs*	39	43	40	37	2.17	37–43	W/AW	2.63	2.85	2.46	2.72	0.14	2.46–2.85
*cs*	22	26	26	21	2.28	21–26	PW/AW	1.78	1.73	1.7	1.82	0.04	1.70–1.82
PaScFed	76	73	70	70	2.49	70–76	PW/L	0.84	0.8	0.86	0.86	0.02	0.80–0.86
PaScGed	65	67	61	60	2.86	60–67	ISD/AP	2.06	1.95	2.11	2.03	0.06	1.95–2.11
GL	136	147	136	141	4.53	136–147	Ti I/Ge I	1.48	1.49	1.56	1.51	0.03	1.48–1.56
Ta I (H)	22	19	23	20	1.58	19–23	Ti II/Ge II	1.81	1.89	1.77	1.84	0.04	1.77–1.89
Ta I (L)	177	180	172	174	3.03	172–180	Ti III/Ge III	1.95	2.09	1.98	2.16	0.08	1.95–2.16
Ti I	298	310	308	307	4.6	298–310	Ti I/AW	6.08	6.46	5.7	6.14	0.27	5.70–6.46
Ge I	201	208	197	203	3.96	197–208	Ti III/AW	8.35	9.08	7.81	8.98	0.51	7.81–9.08
TFe I	133	147	136	141	5.31	136–147	Ti III/Ti I	1.37	1.41	1.37	1.46	0.04	1.37–1.46
BFe I	132	140	133	133	3.2	132–140	Ti II/PW	3.24	3.59	3.14	3.34	0.17	3.14–3.59

## Data Availability

All data are available in this paper.
